# EXPERIENCES USING A SMARTHEALTH SYSTEM: TWO CASES OF DEMENTIA FAMILY CAREGIVERS

**DOI:** 10.1093/geroni/igac059.1659

**Published:** 2022-12-20

**Authors:** Karen Rose, Eunjung Ko, Sooyoung Kim, Kristina Gordon, Hongning Wang, John Stankovic

**Affiliations:** The Ohio State University, Columbus, Ohio, United States; The Ohio State University, Columbus, Ohio, United States; Ohio State University, Columbus, Ohio, United States; The University of Tennessee Department Of Psychology, Knoxville, Tennessee, United States; University of Virginia, Charlottesville, Virginia, United States; University of Virginia, Charlottesville, Virginia, United States

## Abstract

Family caregivers for persons with dementia often times experience stress and burden while caregiving. The COVID-19 pandemic has worsened this situation with increased physical and social isolation, further resulting in health risks in this population. Technology-based interventions have become more commonplace in today’s world to address ongoing caregiver needs although gaps in technological literacy and usage still exist among many users. This case study aims to report on the implementation of an ongoing Smarthealth technology intervention for two older adult family caregivers of persons with dementia and to explore their experiences with this system. Data were collected through acoustic monitoring, survey administration, and semi-structured interviews. Intervention effects on changes in emotional states will be discussed. Study findings showed the intervention improved self-awareness of emotional care and reactions to care recipients. Findings in this study highlight the importance and challenges of real-time technology-based intervention implementation in older adult caregiving populations.

